# Patient stratification by genetic risk in Alzheimer’s disease is only effective in the presence of phenotypic heterogeneity

**DOI:** 10.1371/journal.pone.0310977

**Published:** 2025-01-09

**Authors:** Jack Euesden, Muhammad Ali, Chloe Robins, Praveen Surendran, Padhraig Gormley, for the Alzheimer’s Disease Neuroimaging Initiative (ADNI), David Pulford, Carlos Cruchaga

**Affiliations:** 1 Biostatistics, GSK Pharma R&D, Stevenage, Hertfordshire, United Kingdom; 2 Washington University School of Medicine, NeuroGenomics and Informatics Center, St. Louis, MO, United States of America; 3 Genomic Sciences, GSK Pharma R&D, Collegeville, PA, United States of America; 4 Genomic Sciences, GSK Pharma R&D, Stevenage, Hertfordshire, United Kingdom; 5 Genomic Sciences, GSK Pharma R&D, Cambridge, MA, United States of America; Niigata University, JAPAN

## Abstract

Case-only designs in longitudinal cohorts are a valuable resource for identifying disease-relevant genes, pathways, and novel targets influencing disease progression. This is particularly relevant in Alzheimer’s disease (AD), where longitudinal cohorts measure disease “progression,” defined by rate of cognitive decline. Few of the identified drug targets for AD have been clinically tractable, and phenotypic heterogeneity is an obstacle to both clinical research and basic science. In four cohorts (n = 7241), we performed genome-wide association studies (GWAS) and Mendelian randomization (MR) to discover novel targets associated with progression and assess causal relationships. We tested opportunities for patient stratification by deriving polygenic risk scores (PRS) for AD risk and severity and tested the value of these scores in predicting progression. Genome-wide association studies identified no loci associated with progression at genome-wide significance (α = 5×10^−8^); MR analyses provided no significant evidence of an association between cognitive decline in AD patients and protein levels in brain, cerebrospinal fluid (CSF), and plasma. Polygenic risk scores for AD risk did not reliably stratify fast from slow progressors; however, a deeper investigation found that APOE ε4 status predicts amyloid-β and tau positive versus negative patients (odds ratio for an additional APOE ε4 allele = 5.78 [95% confidence interval: 3.76–8.89], *P*<0.001) when restricting to a subset of patients with available CSF biomarker data. These results provided no evidence for large-effect, common-variant loci involved in the rate of memory decline, suggesting that patient stratification based on common genetic risk factors for progression may have limited utility. Where clinically relevant biomarkers suggest diagnostic heterogeneity, there is evidence that a priori identified genetic risk factors may have value in patient stratification. Mendelian randomization was less tractable due to the lack of large-effect loci, and future analyses with increased samples sizes are needed to replicate and validate our results.

## Introduction

Alzheimer’s disease (AD) is a heritable, progressive neurodegenerative condition. Globally, an estimated 50 million people are living with AD, and this is predicted to triple by 2050 [1]. There are few approved therapies, with only a limited number targeting amyloid-β aggregates, reported to be beneficial in delaying disease progression [2]. The disease is also notable for its clinical heterogeneity in rates of cognitive decline [3, 4]. For these reasons, two research questions are timely and critical: (1) the identification of novel targets for AD drug discovery and (2) testing the value of statistical genetic methods built on genome-wide association studies (GWAS) [5] to unpick the diagnostic and clinical heterogeneity seen amongst AD patients. The heritability of AD and the range of publicly available data mean that an approach driven by genetics is plausible and potentially fruitful.

Statistical genetic methods exploit diseases’ heritability to better understand their etiology and the pathophysiology of individual patients. AD has an estimated heritability as high as 79% from twin studies [6]; that is, almost 80% of the variability in AD status in the population can be attributed to additive genetic factors. The “Common Disease Common Variant” hypothesis [7, 8] posits that a heritable disease that is common within a population must therefore have individual genetic risk factors which are common within a population. The individual genetic variants associated with AD can be elucidated by GWAS, in which millions of individual genetic variants (single nucleotide polymorphisms [SNPs], and insertion/deletion polymorphisms [indels]) can be genotyped or imputed from genotyped data in a sample of cases (diagnosed AD patients) and controls. Logistic regression is then used to identify genetic variants that are more common in cases than controls, thus implicating individual genetic variants, genes, and ultimately proteins in disease pathogenesis. Genome-wide association studies have successfully identified many independent genomic regions, implicating multiple genes associated with risk of AD. Recently, Bellenguez et al. [9] investigated a sample of 111,326 cases (including individuals with a family history of AD) and 677,663 controls to identify 75 independent genomic regions implicated in the etiology of AD risk.

GWAS have primarily studied risk, and most translational applications concern therapy or prognostication for progression, thus GWAS for disease risk might fail to find interesting targets or prognostic markers. Therefore, the rationale for investigating rate of memory decline in AD patients is two-fold. Firstly, there are several diseases in which fast progressors also have a higher burden of disease risk factors. This is not a universal principal, but examples include Huntington’s Chorea, where there is an overlap between high risk and fast progression [10]. This “progression within cases” paradigm has proven fruitful elsewhere, such as within Parkinson’s disease [11, 12]. Secondly, there is the possibility that, given the diagnostic heterogeneity of AD, “cases” may include misdiagnosed individuals who would be expected to show slower disease progression [4]. In either case, studying genetic differences between fast and slow progressors can enable identification of targets that modulate progression in cases and enable cases to be prospectively stratified in future clinical trials.

Recently there have been several larger AD risk GWAS [9, 13, 14]; despite their large sample size, AD is a common phenotype with a late age at onset and, therefore, collecting appropriate controls is challenging. AD can also be challenging to distinguish from other dementias, hampering the collection of cases. Beyond studies of the genetics of disease susceptibility, few have evaluated the genetic architecture of AD progression [15, 16], which could arguably identify novel disease-modifying molecular targets for future development. Identifying risk factors for faster disease progression within AD has focused on GWAS, in a similar paradigm to studying AD case/control. Most recently, Euesden et al. [15] studied a cohort of 1996 AD cases from two clinical trials which had measured cognitive decline in AD over 48 weeks. The authors found no individual genome-wide significant variants associated with rate of cognitive decline but did find a nominal association between rate of progression and polygenic risk score for AD risk, based on the 2013 IGAP AD GWAS [17]. This study had two major limitations. The first was the narrowly defined inclusion/exclusion criteria required for clinical trial cohort data, both in terms of existing duration of illness and also of follow-up time used to measure cognitive decline. The second is the sample size of the GWAS used to prioritize variants for PRS. Here, we brought together the largest longitudinal cohorts of genotyped AD patients to date to perform a GWAS for cognitive decline in AD cases. In addition, given the substantially increased sample sizes available in case-control GWAS for the prioritization of AD risk alleles, the increased sample size available for testing the predictive ability of AD risk PRS, and the opportunity to investigate AD PRS in less narrowly defined cohorts, the present study also sought to expand this paradigm to address the limitations of early disease progression studies. There were two objectives to incorporating AD risk PRS into the study of cognitive decline in AD: firstly, to quantify the relationship between onset (risk) and severity (progression), secondly to stratify individuals to understand heterogeneity and potentially target subgroups in the future.

Finally, traditional statistical genetic methods are increasingly being augmented by the leveraging of large published ‘omics datasets, including protein quantitative trait loci (pQTL) datasets, which can be synthesized alongside GWAS results to provide additional biological context. For instance, Mendelian randomization (MR) methods can integrate pQTL and GWAS summary statistics to test if protein expression has a causal effect on disease, which may aid the identification and prioritization of targets for therapeutics. Thus, our third aim was to augment the target discovery initiative from GWAS with published pQTL datasets. Using MR, we integrated the AD progression GWAS with brain, CSF, and plasma pQTL datasets [18].

## Methods

The study was approved by the ethics committee at every participating institution—Washington University in St. Louis [WUSTL] IRB and Advarra Institutional Review Board for WUSTL and GSK cohorts respectively—and was conducted according to the recommendations of Good Clinical Practice and the Declaration of Helsinki"". All patients provided written informed consent to participate in the study.

### Overview of participants

Participants were included from four cohorts: 2 GSK clinical trials (GSK), Knight Alzheimer’s Disease Research Center (Knight-ADRC–termed “MAP”), Alzheimer’s Disease Neuroimaging Initiative (ADNI), and National Alzheimer’s Coordinating Center (NACC). The GSK cohort consisted of 1842 AD patients from 2 separate phase 3 clinical trials investigating rosiglitazone; these individuals have been described elsewhere [19]. Ethics approval for the genetic analysis of the GSK cohort and oversight were provided by the Advarra Institutional Review Board (sponsor protocol approval notice: Pro00047686). A total of 5399 AD patients with GWAS and longitudinal clinical data were included, where 913, 878, and 3608 participants are derived from the Memory and Aging Project (MAP) at the Knight Alzheimer’s Disease Research Center (Knight-ADRC), Alzheimer’s Disease Neuroimaging Initiative (ADNI), and National Alzheimer’s Coordinating Center (NACC), respectively (**Table 1**). ADNI data was accessed for research purposes on 22-Nov-2021, NACC data was accessed for research purposes on 01-Oct-2021, Knight ADRC data was accessed for research purposes on 29-Dec-2021, GSK data was accessed for research purposes on 18-Feb-2021. Authors did not have access to information that could identify individual participants during or after data collection.

**Table 1:** Overview of demographic properties of each cohort.

Cohort	Sample size	% Female	Mean Age at Baseline (SD)	Mean Years in Education (SD)	Mean weeks to follow-up, averaged across follow-ups (SD)	Average number of Follow-up Visits (SD)
GSK	1842	44.3	73.7 (8.10)	11.2 (3.86)	48 (0)	1 (0)
ADNI	878	41.69	78.61 (7.78)	15.78 (2.84)	216 (133)	3.88 (2.57)
NACC	3608	51.14	80.41 (9.98)	15.93 (6.27)	242 (124)	3.47 (2.20)
Knight-ADRC	913	54.55	83.77 (8.39)	13.95 (3.32)	262 (139)	3.34 (2.27)

ADNI, Alzheimer’s disease neuroimaging initiative; ADRC, Alzheimer’s Disease Research Center; NACC, National Alzheimer’s Coordinating Center; SD, standard deviation.

Patient selection in the ADNI, Knight ADRC and NACC cohorts followed the following paradigm. Briefly, participants were diagnosed as cognitively normal (controls) or AD (cases), based on the clinical dementia rating (CDR-SB) and cognitive assessment performed by experienced clinicians using a semi-structured interview with knowledgeable collateral source and the symptomatic individual in accordance with the Uniform Data Set protocol of the National Alzheimer’s Coordinating Center [20], as well as a detailed neurological examination. The CDR-SB is a five-point scaling system that describes the overall dementia severity for each participant (no dementia = 0, very mild = 0.5, mild = 1, moderate = 2, and severe = 3). Participants with CDR = 0 were considered as controls, and excluded from the analysis, whereas those with CDR > 0 were defined as cases and considered for the present study. Any participant who was missing information about age, sex, education in years, or genetic principal components (PCs) was excluded from the analysis. Following this rationale, we considered 5,399 individuals as clinically defined AD participants from Knight-ADRC, ADNI, and NACC cohorts. The recruitment protocol for the GSK cohort is outlined in the primary study publication [19], but briefly–the cohort comprises of two clinical trials, AVA102670 and AVA102672. These two cohorts planned to recruit patients with a diagnosis of Alzheimer’s disease from at least 150 centers; inclusion criteria included probable Alzheimer’s by NINCDS-ADRDA criteria, genotyping data on APOE, MMSE score 10 to 26 inclusive at Screening corresponding to mild-to-moderate Alzheimer’s disease, Brain CT or MRI scan performed within the past 12 months or at Screening, showing no evidence of any other potential cause of dementia other than Alzheimer’s disease and exclusion included diagnosis of possible, probable, or definite vascular dementia, supported by MRI or CT imaging.

Descriptions of the WUSTL cohorts can be found elsewhere [16]. Ethics approvals for these three cohorts—collectively termed “Consortium”—was obtained from the respective institutional review boards, and research was carried out in accordance with the approved protocols (Washington University in St. Louis [WUSTL] IRB approval 201109148). Written informed consent was obtained from participants or their family members and all participating institutions approved the study.

Data used in the preparation of this article were obtained from the Alzheimer’s Disease Neuroimaging Initiative (ADNI) database. The ADNI was launched in 2003 as a public-private partnership led by principal investigator Michael W. Weiner, MD. The primary goal of ADNI has been to test whether serial magnetic resonance imaging (MRI), positron emission tomography (PET), other biological markers, and clinical and neuropsychological assessment can be combined to measure the progression of mild cognitive impairment and early AD. Details on genotyping methods can be found in the supplemental materials (**S2 File**).

In addition to genotype data, amyloid-β and tau status was assessed by Innotest in the ADNI cohort and is available on a subset of participants. Amyloid-β (Aβ42) and hyperphosphorylated Tau 181 (pTau) biomarker concentrations from CSF samples, assessed by Innotest platform, were used to stratify participants into distinct categories using the AT(N) classification framework [21]. This framework was applied separately for Aβ42 and pTau biomarkers, following established methodologies [22, 23]. In brief, Gaussian mixture models were utilized to partition quantitative Aβ42 and pTau measurements into high (Biomarker positive) and low levels (Biomarker negative). Participants with low CSF Aβ42 and high pTau levels were categorized as amyloid/tau positive (A+T+), indicating elevated plaque and tangle burden in the brain. Conversely, individuals with high Aβ42 and low pTau levels were designated as amyloid/tau negative (A−T−), signifying low plaque and tangle pathology in the brain. Participants exhibiting low CSF Aβ42 and pTau levels were classified as amyloid positive and tau negative (A+T−), representing preclinical stages of AD characterized by elevated plaque but minimal tangle pathology in the brain. Overall, the CSF biomarker data was available for 613 individuals from the ADNI cohort where 329 individuals were categorized as A+T+, 135 as A+T-, 34 as A-T+, and 115 as A-T-.

### Quality control

Imputed genotypes were hard coded to genotype calls, filtered based on MAF>1%, imputation quality R^2^>0.3, HWE p>1x10^−6^. Individuals were retained if genome-wide missingness was <2%, self-reported ethnicity as “White/Caucasian/European” and based on ancestry-informative genetic principal components (PC1 was filtered to be within 3 standard deviation (SD) of its mean, and PC2 filtered to be within 4 SD of its mean in the GSK cohort and 3 SD of its mean in the WUSTL Consortium cohorts).

GSK data was sex, duplicate and cryptic relationship checked as per a pipeline described elsewhere [15]. For non-GSK consortia data, duplication and relatedness of participants were estimated from identity-by-descent (IBD) analysis carried out in Plink version 1.9 [24]. In case of related participants ($\hat{\pi }\geq 0.25$), the samples were prioritized from Knight-ADRC cohort or the ones with a higher number of variants that passed the quality control (QC). Sex identification was verified by analyzing SNPs from the X-chromosome.

### Phenotype definition

The Clinical Dementia Rating-Sum of Boxes (CDR-SB), a measure of cognitive performance where higher scores indicate worse cognitive performance [25], was collected longitudinally in each cohort. To convert each individual’s longitudinal CDR-SB measurements into a single phenotype per individual, the following steps were taken in each cohort separately. A mixed model was fit with an unstructured variance-covariance structure, regressing CDR-SB on time, with random intercepts and slopes for each individual. Each individual’s slope-specific best linear unbiased prediction (BLUP) was then extracted and treated as a phenotype with the interpretation “individual deviation from overall average CDR-SB change per day,” for each individual.

### Genome-wide association studies

GWAS were performed in each cohort separately using linear regression (additive model) implemented in PLINK v2.0.a2.3 [24] adjusted for the first two genetic principal components and the following clinical covariates: baseline CDR-SB, age, gender, years in education, and, for the GSK cohort, trial ID. Meta-analysis was performed using METAL’s standard-error based method [26] as this was considered conservative and makes full use of study-level standard errors (SEs), which were available for all SNPs.

### Polygenic risk scores

To test the prediction of AD genetic risk on cognitive decline in AD amongst cases, polygenic risk scores were calculated using weights and *P* values derived from the Schwartzentruber meta-analysis [14]. This study meta-analyzed a large AD case-control GWAS [27] with a study of individuals with family history of AD, to give a total effective sample size of 63,926 cases and 102,934 controls. PRS were calculated in each cohort separately using PRSice2 [28]. For each analysis, thresholds are tested between 0.0001 and 0.5 at 0.00005 increments (plus an additional threshold at *P* = 1), and the score at the most predictive threshold (lowest *P* value for PRS coefficient) is retained in order to optimize association with the test phenotype (ie, progression), as described elsewhere [29]. PRS were standardized in each cohort separately to have zero mean and unit variance. PRS are aggregated across cohorts at each *P* value threshold separately by meta-analyzing the regression coefficients and standard errors in each cohort, using a standard error–based method to match the one implemented by METAL.

### Mendelian randomization

Potential causal relationships between individual proteins and AD progression, focusing on proteins in plasma, brain, and CSF, were identified using MR methods and the TwoSampleMR R package [30]. These MR analyses combined summary statistics from the AD progression GWAS with previously published brain, CSF, and plasma pQTL datasets [18]. For each tissue with pQTL data, MR analyses were restricted to proteins with cis-pQTLs (*P*<5x10^-8^) that were included in the AD progression GWAS and could be selected as instrumental variables (IVs). Instrumental variables were selected for each protein by performing linkage disequilibrium (LD)–clumping of the cis-pQTLs using the PLINK method implemented in TwoSampleMR with a default r^2^ threshold of 0.001. The effect of each protein on AD progression was estimated using the Wald ratio method for proteins with a single IV and the inverse-variance weighted (IVW) regression method for proteins with multiple IVs. Multiple testing correction was performed using a Bonferroni threshold based on the total number of tests performed.

### Heritability analysis

Linkage disequilibrium score regression (LDSC) [31] was used to estimate the heritability of cognitive decline in AD using the main meta-analysis. The bivariate coheritability between this and AD risk, based on the Schwartzentruber [14] GWAS, could also be calculated.

## Results

### Demographic data

Following QC, 7241 individuals remained across the four cohorts and were available for subsequent GWAS analysis. The demographic composition of the cohorts is shown in **Table 1**.

### GWAS of cognitive decline

**Fig 1:**
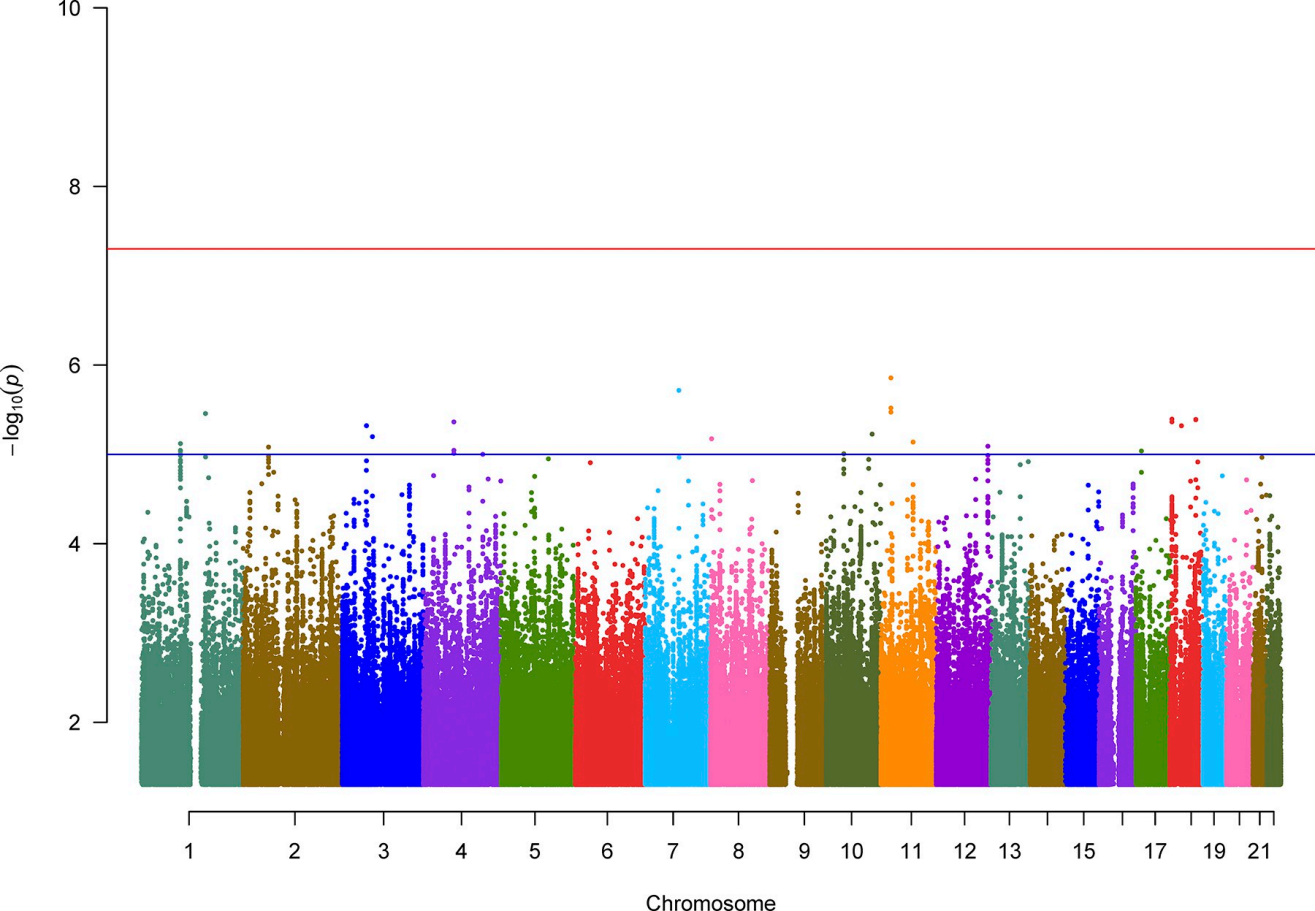
Manhattan plot of meta-analysis across 4 cohorts. No individual genetic variants reached genome-wide significance (α = 5x10^−8^). Suggestively significant associations (*P*<10^−5^) are highlighted in **S3 Table in S1 Data**.

Following QC, 7,454,167 variants remained for analysis (**S1 Fig in S4 File**). With a response phenotype of cognitive decline, defined as difference in CDR-SB change-per-day from the cohort average (**S2 Fig in S4 File**), no individual variants reached genome-wide significance in any of the four individual cohorts or in the main meta-analysis (**Fig 1**). Of the 75 GWAS significant variants identified in Bellenguez et al. [9], 68 were present in our final meta-analysis; of these, 13 variants were nominally associated (*P*<0.05) but no variants had *P*<0.001 in the 4-cohort meta-analysis (**S1 Table in S1 Data**). Amongst the top 100 variants identified in the meta-analysis GWAS (**S2 Table in S1 Data**), none have previously been associated with AD in case-control GWAS. A variant in *BIN1* (rs6733839) has been previously associated with AD risk [9] and in this meta-analysis, a synonymous variant, rs61748155, located within *BIN1* but not previously associated with AD risk, showed a suggestive *P* value for association with cognitive decline in AD (*P* = 3.2×10^−5^). There was no LD between rs6733839 and rs61748155 (R^2^<0.05). Although the sample size for this GWAS is still relatively modest compared to AD susceptibility GWAS [9], it nonetheless represents the largest genotyped cohort with longitudinal data in which to study AD progression to date and is larger than previous progression GWAS in neurodegeneration, some of which that have reported genome-wide significant loci (eg, in Parkinson’s disease [11] and Huntington’s Chorea [32]).

### Heritability analysis

The estimate of heritability (h^2^_g,_ ie, SNP heritability, the portion of narrow-sense heritability captured by common genetic variants) for cognitive decline in AD is estimated via LDSC; this summary statistic approach will have comparable statistical power to the genotype-based approach GREML. h^2^g was estimated as 0.0452 (SE = 0.0545); we exclude true h^2^_g_ above 15.2% with 95% confidence based on an asymptotic approximation. These results are compatible with heritability for cognitive decline in AD having a heritable component comparable with AD risk as estimated in Schwartzentruber et al. [14]–h^2^_g_ = 0.0711 (SE = 0.0113) or Parkinson’s disease risk (ie, case control) estimated by Nalls et al. [33]–h^2^_g_ = 0.22 (95% CI: 0.18–0.26). They are also compatible with cognitive decline having minimal heritability and therefore are challenging to interpret alone.

The bivariate coheritability (genetic correlation) between AD risk and cognitive decline in AD was estimated at −0.0382 (SE = 0.2508). The lack of a statistically significant genetic overlap between risk and progression is consistent with findings from LD score regression in Parkinson’s disease [11].

### Polygenic risk scores

**Fig 2:**
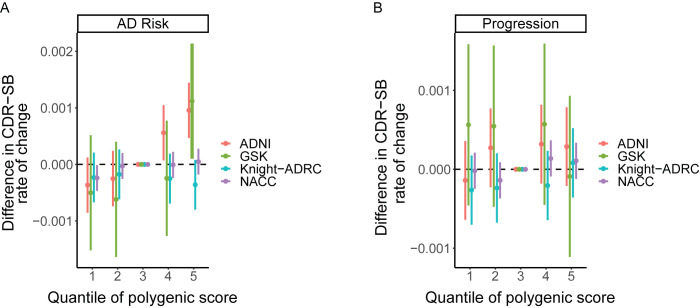
Average CDR-SB change in patients stratified into 5 PRS quantiles, compared to patients in middle quantile. PRS is calculated for risk of two separate phenotypes, and cognitive decline is regressed on this; each of 4 cohorts are shown separately. **(A)** PRS for AD risk based on Schwarzentruber et al.; **(B)** PRS for cognitive decline based on GWAS results from the present study. AD, Alzheimer’s disease; ADNI, Alzheimer’s disease neuroimaging initiative; ADRC, Alzheimer’s Disease Research Center; CDR-SB, Clinical Dementia Rating-Sum of Boxes; GWAS, genome-wide association study; NACC, National Alzheimer’s Coordinating Center; PRS, polygenic risk score; SE, standard error; WUSTL, Washington University in St. Louis.

AD risk PRS is not associated with cognitive decline amongst diagnosed AD patients; meta-analyzing across the four cohorts, the association between PRS for AD risk and progression was not statistically significant (meta *P* = 0.0011) at a standard PRS significance threshold of 0.001; it is important to account for the actual multiplicity burden introduced by deriving and testing PRS at a large range of potential thresholds, across which there will be a degree of correlation and therefore we chose a significance threshold of P<0.001 based on simulations performed in previous published literature [29] (**Fig 2A; Table 2**).

**Table 2:** PRS predicting cognitive decline at the most predictive threshold in each cohort separately and following meta-analysis. “AD Risk” is the predictive ability of PRS derived from Schwartzentruber et al. 2021. “Progression” is the predictive ability of PRS derived from the progression GWAS reported here; ie, the GSK GWAS is used to construct PRS for the WUSTL cohorts, and a meta-analysis of the 3 WUSTL cohorts is used to construct PRS for the GSK cohort.

Analysis	Type	cohort	P-value Threshold for SNP inclusion in PRS	Additional variance explained by PRS	*P*	Coefficient	SE	Number of SNPs included in best-fitting PRS
**AD Risk **	**Individual Cohort**	**GSK**	1.00×10^−4^	4.39×10^−3^	4.15×10^−3^	4.73×10^−4^	1.65×10^−4^	178
		**ADNI**	2.00×10^−4^	1.71×10^−2^	8.60×10^−6^	3.50×10^−4^	7.82×10^−5^	711
		**Knight-ADRC**	1	2.61×10^−3^	1.02×10^−1^	−8.67×10^−5^	5.29×10^−5^	268,264
		**NACC**	1.6355×10^−1^	1.53×10^−3^	1.48×10^−2^	1.43×10^−4^	5.88×10^−5^	90,616
	**Meta-Analysis**	**4-cohort meta-analysis**	4.55×10^−3^	NA	1.05×10^−3^	1.11×10^−4^	3.40×10^−5^	NA
**Progression**	**Individual Cohort**	**GSK**	1.05×10^−3^	2.04×10^−3^	5.08×10^−2^	−3.20×10^−4^	1.64×10^−4^	423
		**ADNI**	5.65×10^−3^	1.53×10^−3^	1.85×10^−1^	9.52×10^−5^	7.17×10^−5^	2291
		**Knight-ADRC**	1.45×10^−3^	3.01×10^−3^	7.86×10^−2^	1.21×10^−4^	6.86×10^−5^	624
		**NACC**	1.50×10^−4^	1.26×10^−3^	2.74×10^−2^	7.79×10^−5^	3.53×10^−5^	71
	**Meta-Analysis**	**Meta-analysis of 3 WUSTL cohorts**	5.50×10^−4^	NA	2.69×10^−2^	6.41×10^−5^	2.90×10^−5^	NA

AD, Alzheimer’s disease; ADNI, Alzheimer’s disease neuroimaging initiative; ADRC, Alzheimer’s Disease Research Center; GWAS, genome-wide association study; NA, not available; NACC, National Alzheimer’s Coordinating Center; PRS, polygenic risk score; SE, standard error; WUSTL, Washington University in St. Louis.

Similarly, AD cognitive decline PRS is not significantly associated with cognitive decline in this study; the association between GSK GWAS and progression in the WUSTL Consortium cohort (meta *P* = 0.051), and between the WUSTL Consortium 3-cohort meta-analysis and progression in the GSK cohort (*P* = 0.027) were not statistically significant (**Fig 2B; Table 2**), illustrating a lack of clinical utility in these sample sizes.

### The influence of genetic risk for AD within the ADNI cohort

Investigating quantiles of polygenic scores differentiating fast from slow progressors showed that CDR-SB rate of change was generally not significantly higher in high PRS individuals than median PRS individuals, nor was it significantly lower in low PRS individuals than median PRS individuals (**S3 Table in S1 Data, Fig 2)**. Exceptions to this were found in the lowest quantile patients in NACC who progress slower than average (*P* = 0.04), and, progressing faster than average, the second highest quantile in ADNI (*P* = 0.025) and the highest quantiles in ADNI (*P* = 0.0001) and GSK (*P* = 0.031). As this relationship is strongest in ADNI, which has biomarker data available, additional follow-up analyses were performed in ADNI only to dissect the clinical and genomic factors driving this association.

PRS was recalculated in ADNI excluding the APOE gene region (43907927–45908821 base pairs on chromosome 19 in GRCh38). Following the approach used above for PRS, the *P* value threshold for selecting SNPs was identified to minimize the *P* value for PRS predicting progression; at this most predictive threshold, PRS did not significantly predict AD progression in ADNI (*P* = 0.051), however the coefficient for this PRS excluding the APOE gene region trends in the same direction as the genome-wide PRS. APOE ε4 allele count alone showed a significant association with progression in ADNI (*P* = 1.65×10^−8^, **S4 Table in S1 Data, S3 Fig in S4 File**). These two results suggest that APOE ε4 allele status is driving the association between PRS and progression in ADNI, with nominal evidence for a genome-wide contribution.

ADNI had biomarker data available (CSF tau and amyloid-β), and so this relationship was investigated further. Analysis was restricted to individuals with Innotest data who were either double (amyloid and tau) biomarker positive or double biomarker negative. A logistic model was fit to this data to quantify the effect of APOE ε4 allele on biomarker status.

**Fig 3:**
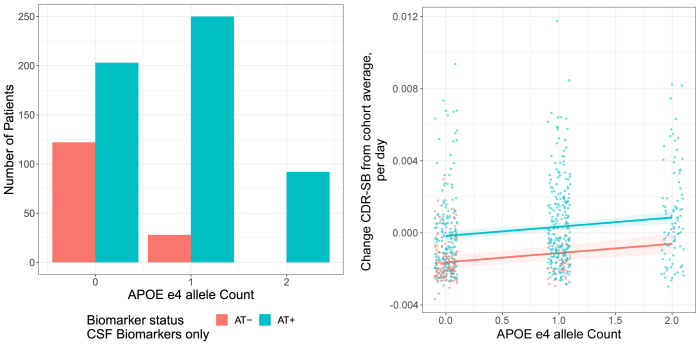
APOE ε4 allele count, Biomarker status and cognitive decline in ADNI: The relationship between APOE ε4 allele count and cognitive decline (CDR-SB change) in ADNI, split by biomarker status (AT+ = amyloid-β and tau positive; AT- = amyloid-β and tau negative). **(A)** Biomarker positivity was significantly higher in APOE ε4 allele carriers; **(B)** cognitive decline was significantly faster in biomarker-positive patients. NB, only individuals with AT+ or AT− data included. ADNI, Alzheimer’s disease neuroimaging initiative; CDR-SB, Clinical Dementia Rating-Sum of Boxes; CSF, cerebrospinal fluid.

Within the ADNI cohort, n = 444 participants had data on both amyloid-β and tau. Of these, n = 329 were amyloid-β and tau positive (AT+), and n = 115 were amyloid-β and tau negative (AT−) (**Fig 3A**). Looking within these individuals alone, a one-unit increase in APOE ε4 allele count had an odds ratio of 5.78 (95% CI: 3.76–8.89, *P* = 1.23×10^−15^) on biomarker positivity (**Fig 3A**) in a logistic model adjusting for the same covariates as in the original GWAS.

Regressing progression on both biomarker status and APOE ε4 allele count, adjusting for the same covariates as in the original GWAS, the interaction between biomarker status and APOE ε4 allele count was not statistically significant (*P* = 0.3). With the caveat that there is a high correlation between APOE ε4 allele count and biomarker status, a one-allele increase in APOE ε4 allele count was associated with significantly faster progression (*P* = 0.0015, CDR-SB was 3.72 × 10^−4^ higher per day on study, 95% CI: 1.43 × 10^−4^–6.02 × 10^−4^). Progression was faster in amyloid-β and tau positive versus amyloid-β and tau-negative patients (*P* = 1.46 × 10^−7^, CDR-SB was 1.03 × 10^−3^ higher per day on study, 95% CI: 6.51 × 10^−4^ −1.42 × 10^−3^) (**Fig 3B**).

### Mendelian randomization

**Fig 4:**
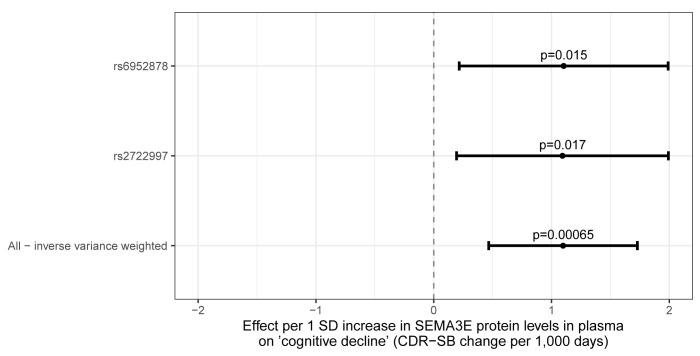
MR results. The MR estimate of the causal effect of plasma SEMA3E protein levels on cognitive decline is presented with 95% confidence intervals. “Cognitive decline” is here defined as change in CDR-SB per 1,000 days as a deviation from the cohort-average CDR-SB trajectory. The two listed variants were used as instrumental variables (IVs) for the MR test using the IVW method. IVW, inverse-variance weighted; MR, Mendelian randomization; SD, standard deviation.

Mendelian randomization was performed to identify potential causal proteins using multitissue pQTL data and the meta-analysis summary statistics for rate of AD progression. In this analysis, a GWAS phenotype of ‘CDR-SB change from cohort average per-1000 days’ was used in order to mitigate rounding issues. For each protein, independent cis-pQTLs were selected as instrumental variables (IV) (r^2^<0.001, *P*<5 × 10^−8^). No proteins measured in CSF, brain, or plasma were found to have a significant causal effect on cognitive decline at a Bonferroni-adjusted significance threshold (*P*<2.2x10^−4^, α = 0.05/219 tests performed). The strongest nominal MR result suggests that plasma Semaphorin 3E (SEMA3E) levels, as predicted by genotype, may be associated with AD progression (*P* = 6.5x10^−4^) and indicates that an increase in plasma SEMA3E levels associates with increased cognitive decline or more rapid AD progression (**Fig 4**). The plasma SEMA3E MR test used two independent IVs, rs2722997 and rs6952878, and the IVW regression method. Both the IVs were strongly associated with SEMA3E protein levels in plasma (*P*<2x10^−15^), but only nominally associated with cognitive decline (*P*<0.02). Future analyses using increased sample sizes and having increased power are needed to validate this suggestive plasma SEMA3E MR result.

## Discussion

There were two principal aims to the present study: target discovery and patient stratification. Target discovery sought to identify genes and proteins in which genetic variation correlates with speed of progression amongst AD patients. This was tested by performing the largest GWAS to date of disease progression, specifically, the rate of cognitive decline within AD patients, and augmenting the results of this by incorporating pQTL analyses through Mendelian randomization. Patient stratification sought to test whether genetic risk factors, either for AD risk or for cognitive decline within AD, can be used to stratify meaningfully a phenotypically heterogeneous cohort of AD patients.

The target discovery initiative presented here incorporated the largest GWAS (n = 7241) to date of cognitive decline in AD and is unique not just in its size but in the methodological harmonization (QC, phenotype definition) that was possible in this collaboration. Across four cohorts, these results do not provide evidence for any individual large-effect loci in the modulation of cognitive decline within AD patients and differs somewhat to findings in other similarly sized progression GWAS in Parkinson’s disease where several loci were recently associated with mortality, cognitive impairment, and motor progression [12]. Heritability estimates for progression were low, thus failing to provide evidence for a high (>20% or more) heritability for AD progression, based on common genetic variants. Whilst the heritability of AD progression is not statistically significant, a point estimate of 4.5% does not necessarily preclude the future discovery of genetic variants influencing the rate of cognitive decline in AD with the use of larger sample sizes. We note that LDSC performed on summary statistics- rather than a per-cohort raw-data method such as GCTA—has the advantage of a single analysis on the largest available GWAS, this must be interpreted in the context of recent findings that LDSC heritability estimates may be biased downwards relatively to corresponding estimates from GCTA [34]. Furthermore, the low SNP heritability does not rule out a meaningful contribution from other sources such as rare variants, epistatic factors, or copy number variants.

MR analyses incorporated pQTL and GWAS data–proteins are more common druggable molecular targets than mRNAs, therefore pQTL MR analyses, rather than other MR analyses such as eQTL, would best aid drug discovery and prioritization. This analysis identified a relationship between a signalling molecule involved in neuronal development—SEMA3E—and cognitive decline. This analysis provided suggestive evidence that higher levels of plasma SEMA3E associates with higher rates of cognitive decline–which is inconsistent with literature that SEMA3E may be critical for normal hippocampal development [35] and that together with its receptor, PLXDN1, may have a neuroprotective role [36]. While there is an established association between SEMA3E and CHARGE syndrome [37], more recent GWAS suggest that variants in the SEMA3E locus are associated with neurological and psychiatric phenotypes [38]. While our data provide initial evidence and may suggest that increased levels of plasma SEMA3E is associated with increased rate of cognitive decline in AD patients, they should be treated with caution. Importantly, these analyses were hampered by the low power of the progression GWAS, reinforcing the need to validate results in progression GWAS with an even larger sample size. Nevertheless, these results do provide initial biological insights amongst small-effect loci that are consistent with emerging hypotheses for the neurobiology of cognitive decline in AD.

Genome-wide heritability analyses ought to be used in the future to prospectively assess the tractability of target discovery and patient stratification initiatives. The present findings (no genome-wide significant loci and poor patient stratification from PRS) are consistent with the low SNP heritability calculated here for cognitive decline in AD and the low bivariate heritability between progression and risk and indicate that concerns around using risk PRS to predict progression [39] may not be confounding the current analysis. While this study does not support patient stratification amongst AD patients, either when using PRS for AD risk or PRS for cognitive decline in AD, the findings here do not preclude using PRS for predicting patient severity in other phenotypes, even in a clinical trial setting [40].

The conclusions of the present study must be interpreted in the context of several key limitations. Firstly, CDR-SB score is bounded by 0 and 18 and individual patients’ CDR-SB score change is bounded by a decrease of 18 and an increase to the value of their baseline score. This saturation could create floor/ceiling effects and potentially have implications for statistical power. An analysis method has not been used that is sensitive to this. Secondly, CDR-SB measures dementia severity generally, not solely cognitive decline–in the within-patients context of this paradigm these are assumed to be interchangeable, but this could be further investigated using deeper phenotyped data with additional granularity on neuropsychological domains. Additional granularity in future prospective cohorts will also integrate data on duration of disease in order to determine whether fast progressors are early-disease individuals.

The separation of cases in ADNI is perhaps the most interesting and useful finding presented here, and it can be understood in the context of the potentially considerable clinical heterogeneity present in the ADNI cohort. That is to say, an admixture of true AD cases with other dementia cases. We were able to disentangle this using genetic data, ie, APOE ε4 allele count. This is consistent with results elsewhere that APOE ε4 allele count correlates with AD biomarker levels [41]. Thus, the performance of APOE ε4 allele count for patient stratification may appear inflated in this setting as the present analysis focused on the subset of ADNI cases who also have biomarker data available, and who have clear biomarker evidence for being true cases (amyloid-β+ tau+) or for being misdiagnosed cases (amyloid-β− tau−). Nevertheless, this provides evidence that genetic information more generally may have a fruitful role in patient stratification in cases where diagnostic heterogeneity is potentially high and may harm statistical power by diluting any signal.

## Conclusions

In the largest GWAS to date, there is no evidence for large genetic effects driving phenotypic heterogeneity in AD cases in terms of cognitive decline; however, augmenting this analysis with additional proteomic data implicates proteins that may have plausible roles in AD pathophysiology. Here, we also show that cognitive decline in AD has a low heritability and patient stratification amongst AD cases and that using genetic data is not a viable strategy. Finally, genomically-driven patient stratification techniques may be a valuable tool in the future to screen patients for case-only designs, especially in phenotypes with high heritability or diagnoses which often rely on postmortem data.

## Supporting information

S1 Data (XLSX)

S1 File Acknowledgment list for ADNI publications. The Data and Publications Committee, in keeping with the publication policies adopted by the ADNI Steering Committee, here provide lists for standardized acknowledgement. The list consists of three parts: I. ADNI Infrastructure Investigators and Site Investigators, II. DOD ADNI Infrastructure Investigators and Site Investigators and III. ADNI Depression Infrastructure Investigators and Site Investigators. Infrastructure Investigators represent the names responsible for leadership and infrastructure. Site Investigators represent the names of individuals at each recruiting site. All papers, including methodological papers, should have an acknowledgement list that consists of Infrastructure Investigators plus the FULL list.
(PDF)

S2 File Supplementary methods. Full details on genotyping methods can be found here.
(DOCX)

S3 File Supplementary detail on data availability. Full details on availability of data from each cohort can be found here.
(DOCX)

S4 File (DOCX)

## References

[1] ZhangX-X, TianY, WangZ-T, MaY-H, TanL, YuJ-T. The Epidemiology of Alzheimer’s disease modifiable risk factors and prevention. J Prev Alzheimers Dis. 2021;8(3):313–321. doi: 10.14283/jpad.2021.15 34101789

[2] Haeberlein SB, Aisen PS, Barkhof F, Chalkias S, Chen T, Cohen S, et al. Two Randomized Phase 3 Studies of Aducanumab in Early Alzheimer’s Disease. J Prev Alzheimers Dis. 2022;9(2):197–210. doi: 10.14283/jpad.2022.30 35542991

[3] Beach TG, Monsell SE, Phillips LE, Kukull W. Accuracy of the clinical diagnosis of Alzheimer disease at National Institute on Aging Alzheimer Disease Centers, 2005–2010. J Neuropathol Exp Neurol. 2012;71(4):266–273. doi: 10.1097/NEN.0b013e31824b211b 22437338 PMC3331862

[4] Gaugler JE, Ascher-Svanum H, Roth DL, Fafowora T, Siderowf A, Beach TG. Characteristics of patients misdiagnosed with Alzheimer’s disease and their medication use: an analysis of the NACC-UDS database. BMC Geriatr. 2013;13:137. doi: 10.1186/1471-2318-13-137 24354549 PMC3878261

[5] Morris AP, Cardon LR. Genome‐Wide Association Studies. In: Balding D, Moltke I, Marioni J, editors. Handbook of Statistical Genomics: Two Volume Set. Wiley; 2019. pp. 597–550.

[6] Gatz M, Reynolds CA, Fratiglioni L, Johansson B, Mortimer JA, Berg S, et al. Role of genes and environments for explaining Alzheimer disease. Arch General Psychiatry. 2006;63(2):168–174. doi: 10.1001/archpsyc.63.2.168 16461860

[7] Corvin A, Craddock N, Sullivan PF. Genome-wide association studies: a primer. Psychol Med. 2010;40(7):1063–1077. doi: 10.1017/S0033291709991723 19895722 PMC4181332

[8] Uher R. The role of genetic variation in the causation of mental illness: an evolution-informed framework. Mol Psychiatry. 2019;14(12):1072–1082. doi: 10.1038/mp.2009.85 19704409

[9] Bellenguez C, Küçükali F, Jansen IE, Kleineidam L, Moreno-Grau S, Amin N, et al. New insights into the genetic etiology of Alzheimer’s disease and related dementias. Nat Gen. 2022;54(4):412–436. doi: 10.1038/s41588-022-01024-z 35379992 PMC9005347

[10] Ranen NG, Stine OC, Abbott MH, Sherr M, Codori AM, Franz ML, et al. Anticipation and instability of IT-15 (CAG)n repeats in parent-offspring pairs with Huntington disease. Am J Hum Gen. 1995;57(3):593–602. 7668287 PMC1801258

[11] Tan MMX, Lawton MA, Jabbari E, Reynolds RH, Iwaki H, Blauwendraat C, et al. Genome-wide association studies of cognitive and motor progression in Parkinson’s disease. Mov Disord. 2021;36(2):424–433. doi: 10.1002/mds.28342 33111402 PMC9053517

[12] Tan MMX, Lawton MA, Pollard M, Brown E, Bekadar S, Jabbari E, et al. Genome-wide determinants of mortality and clinical progression in Parkinson’s disease. medRxiv [Preprint]. 2022 [Posted 2023 June 9]. Available from: https://www.medrxiv.org/content/10.1101/2022.07.07.22277297v2 doi: 10.1101/2022.07.07.22277297

[13] de Rojas I, Moreno-Grau S, Tesi N, Grenier-Boley B, Andrade V, Jansen IE, et al. Common variants in Alzheimer’s disease risk stratification by polygenic risk scores. Nat Commun. 2021;12(1):3417. doi: 10.1038/s41467-021-22491-8 34099642 PMC8184987

[14] Schwartzentruber J, Cooper S, Liu JZ, Barrio-Hernandez I, Bello E, Kumasaka N, et al. Genome-wide meta-analysis, fine-mapping and integrative prioritization implicate new Alzheimer’s disease risk genes. Nat Gen. 2021;53(3):392–402. doi: 10.1038/s41588-020-00776-w 33589840 PMC7610386

[15] Euesden J, Gowrisankar S, Qu AX, Jean PS, Hughes AR, Pulford DJ. Cognitive decline in Alzheimer’s disease: Limited clinical utility for GWAS or polygenic risk scores in a clinical trial setting. Genes. 2020;11(5):501. doi: 10.3390/genes11050501 32370229 PMC7290959

[16] Del-Aguila JL, Fernández MV, Schindler S, Ibanez L, Deming Y, Ma S. Assessment of the genetic architecture of Alzheimer’s disease risk in rate of memory decline. J Alzheimers Dis. 2018;62(2):745–756. doi: 10.3233/JAD-170834 29480181 PMC5989565

[17] Lambert JC, Ibrahim-Verbaas CA, Harold D, Naj AC, Sims R, Bellenguez C, et al. Meta-analysis of 74,046 individuals identifies 11 new susceptibility loci for Alzheimer’s disease. Nat Gen. 2013;45(12):1452–1458. doi: 10.1038/ng.2802 24162737 PMC3896259

[18] Yang C, Farias FHG, Ibanez L, Suhy A, Sadler B, Fernandez MV, et al. Genomic atlas of the proteome from brain, CSF and plasma prioritizes proteins implicated in neurologic disorders. Nat Neurosci. 2021;24(9):1302–1312. doi: 10.1038/s41593-021-00886-6 34239129 PMC8521603

[19] Harrington C, Sawchak S, Chiang C, Davies J, Donovan C, Saunders AM, et al. Rosiglitazone does not improve cognition or global function when used as adjunctive therapy to AchE inhibitors in mild-to-moderate Alzheimer’s disease: two phase 3 studies. Curr Alzheimer Res. 2011;8(5):592–606. doi: 10.2174/156720511796391935 21592048

[20] Morris JC, Weintraub S, Chui HC, Cummings J, Decarli C, Ferris S, et al. The Uniform Data Set (UDS): clinical and cognitive variables and descriptive data from Alzheimer Disease Centers. Alzheimer Dis Assoc Disord. 2006 Oct-Dec;20(4):210–6. doi: 10.1097/01.wad.0000213865.09806.92 . 17132964

[21] Jack CR Jr, Bennett DA, Blennow K, Carrillo MC, Feldman HH, Frisoni GB, et al. A/T/N: An unbiased descriptive classification scheme for Alzheimer disease biomarkers. Neurology. 2016 Aug 2;87(5):539–47. doi: 10.1212/WNL.0000000000002923 Epub 2016 Jul 1. ; PMCID: PMC4970664. 27371494 PMC4970664

[22] Ali M, Sung YJ, Wang F, Fernández MV, Morris JC, Fagan AM, et al. Leveraging large multi-center cohorts of Alzheimer disease endophenotypes to understand the role of Klotho heterozygosity on disease risk. PLoS One. 2022 May 26;17(5):e0267298. doi: 10.1371/journal.pone.0267298 ; PMCID: PMC9135221. 35617280 PMC9135221

[23] Timsina J, Gomez-Fonseca D, Wang L, Do A, Western D, Alvarez I, et al. Comparative Analysis of Alzheimer’s Disease Cerebrospinal Fluid Biomarkers Measurement by Multiplex SOMAscan Platform and Immunoassay-Based Approach. J Alzheimers Dis. 2022;89(1):193–207. doi: 10.3233/JAD-220399 Erratum in: J Alzheimers Dis. 2023;91(2):911. ; PMCID: PMC9562128. 35871346 PMC9562128

[24] Chang CC, Chow CC, Tellier LC, Vaatikuti S, Purcell SM, Lee JJ. Second-generation PLINK: rising to the challenge of larger and richer datasets. Gigascience. 2015;4(1):7. doi: 10.1186/s13742-015-0047-8 25722852 PMC4342193

[25] Williams MM, Storandt M, Roe CM, Morris JC. Progression of Alzheimer’s disease as measured by Clinical Dementia Rating Sum of Boxes scores. Alzheimers Dement. 2013;9(Suppl 1):S39–S44. doi: 10.1016/j.jalz.2012.01.005 22858530 PMC3660405

[26] Willer CJ, Li Y, Gonçalo RA. METAL: fast and efficient meta-analysis of genomewide association scans. Bioinformatics. 2010;26(17):2190–2191. doi: 10.1093/bioinformatics/btq340 20616382 PMC2922887

[27] Kunkle BW, Grenier-Boley B, Sims R, Bis JC, Damotte V, Naj AC, et al. Genetic meta-analysis of diagnosed Alzheimer’s disease identifies new risk loci and implicates Aβ, tau, immunity and lipid processing. Nat Gen. 2019;51(3):414–430. doi: 10.1038/s41588-019-0358-2 30820047 PMC6463297

[28] Choi SW, O’Reilly PF. PRSice-2: Polygenic Risk Score software for biobank-scale data. Gigascience. 2019;8(7):giz082. doi: 10.1093/gigascience/giz082 31307061 PMC6629542

[29] Euesden J, Lewis CM, O’Reilly PF. PRSice: polygenic risk score software. Bioinformatics. 2015;31(9):1466–1468. doi: 10.1093/bioinformatics/btu848 25550326 PMC4410663

[30] Hemani G, Zheng J, Elsworth B, Wade KH, Haberland V, Baird D, et al. The MR-Base platform supports systematic causal inference across the human phenome. Elife. 2018;7:e34408. doi: 10.7554/eLife.34408 29846171 PMC5976434

[31] Bulik-Sullivan BK, Loh P-R, Finucane HK, Ripke S, Yang J, Schizophrenia Working Group of the Psychiatric Genomics Consortium; Nick Patterson, et al. LD Score regression distinguishes confounding from polygenicity in genome-wide association studies. Nat Gen. 2015;47(3):291–295. doi: 10.1038/ng.3211 25642630 PMC4495769

[32] Moss DJH, Pardiñas AF, Langbehn D, Lo K, Leavitt BR, Roos R, et al. Identification of genetic variants associated with Huntington’s disease progression: a genome-wide association study. Lancet Neurol. 2017;16(9):701–711. doi: 10.1016/S1474-4422(17)30161-8 28642124

[33] Nalls MA, Blauwendraat C, Vallerga CL, Heilbron K, Bandres-Ciga S, Chang D, et al. Identification of novel risk loci, causal insights, and heritable risk for Parkinson’s disease: a meta-analysis of genome-wide association studies. Lancet Neurol. 2019;18(12):1091–1102. doi: 10.1016/S1474-4422(19)30320-5 31701892 PMC8422160

[34] de la Fuente J, Grotzinger AD, Marioni RE, Nivard MG, Tucker-Drob EM. Integrated analysis of direct and proxy genome wide association studies highlights polygenicity of Alzheimer’s disease outside of the APOE region. PLoS Genet. 2022 Jun 3;18(6):e1010208. doi: 10.1371/journal.pgen.1010208 ; PMCID: PMC9200312. 35658006 PMC9200312

[35] Agata M, Gil V, Pérez-Clausell J, Dasilva M, González-Calixto MC, Soriano E, et al. New functions of Semaphorin 3E and its receptor PlexinD1 during developing and adult hippocampal formation. Sci Rep. 2018;8(1):1–16. doi: 10.1038/s41598-018-19794-0 29358640 PMC5777998

[36] Cariboni A, André V, Chauvet S, Cassatella D, Davidson K, Caramello A, et al. Dysfunctional SEMA3E signaling underlies gonadotrophin-releasing hormone neuron deficiency in Kallmann syndrome. J Clin Invest. 2015;125:2413–2428. doi: 10.1172/JCI78448 25985275 PMC4497752

[37] Källén K, Robert E, Mastroiacovo P, Castilla EE, Källén B. CHARGE Association in newborns: a registry-based study. Teratology. 1999;60(6):334–343. doi: 10.1002/(SICI)1096-9926(199912)60:6&lt;334::AID-TERA5&gt;3.0.CO;2-S 10590394

[38] Churchhouse C, Neale B. RAPID GWAS OF THOUSANDS OF PHENOTYPES FOR 337,000 SAMPLES IN THE UK BIOBANK. 2017 Sep 20 [cited 29 Aug 2023]. In: Neale Lab [Internet]. Available from: http://www.nealelab.is/blog/2017/7/19/rapid-gwas-of-thousands-of-phenotypes-for-337000-samples-in-the-uk-biobank

[39] Mitchell RE, Hartley AE, Walker VM, Gkatzionis A, Yarmolinsky J, Bell JA, et al. Strategies to investigate and mitigate collider bias in genetic and Mendelian randomisation studies of disease progression. PloS Genetics. 2023;19.2:e1010596. doi: 10.1371/journal.pgen.1010596 36821633 PMC9949638

[40] Santoro ML, Ota V, de Jong S, Noto C, Spindola LM, Talarico F, et al. Polygenic risk score analyses of symptoms and treatment response in an antipsychotic-naive first episode of psychosis cohort. Transl Psychiatry. 2018;8(1):174. doi: 10.1038/s41398-018-0230-7 30171181 PMC6119191

[41] Ibanez L, Bahena JA, Yang C, Dube U, Farias FHG, Budde JP, et al. Functional genomic analyses uncover APOE-mediated regulation of brain and cerebrospinal fluid beta-amyloid levels in Parkinson disease. Acta Neuropathol Commun. 2020;8(1):196. doi: 10.1186/s40478-020-01072-8 33213513 PMC7678051

