# Supplementary material for: Patient stratification by genetic risk in Alzheimer’s disease is only effective in the presence of phenotypic heterogeneity
Source: PLoS One. 2025 Jan 9;20(1):e0310977. doi: 10.1371/journal.pone.0310977 (PMC11717250; doi:10.1371/journal.pone.0310977)
Supplement: S2 File — Full details on genotyping methods can be found here. (DOCX) [file pone.0310977.s003.docx]

# *Supplementary Methods*

##

## Methods S1. Details on genotyping.

### GSK

Venous blood was collected from each patient who provided written informed consent for genetic research. Genomic DNA was extracted from peripheral blood using the Gentra Puregene kit on the Autopure LS (Qiagen, Valencia, CA) by Quest Diagnostics (Valencia, CA, USA or Heston, UK). Genotyping was performed using the Affymetrix Axiom PMRA (Santa Clara, CA) by the Bioprocessing Solutions Alliance (BSA) under Brooks Automation Inc. (Piscataway, NJ). The array data with a reference set of haplotypes was used to impute variants across the genome including the HLA region.

The genome-wide array data were used to impute gene dosages. For each array, genotypes were aligned to the reference strand and phased by chromosome using sequence and genotype data to estimate haplotypes and unobserved genotypes with HAPI-UR v1.01 (Williams et al 2012). The phased haplotypes were used to impute genotype dosages using the 1000 Genomes Project reference haplotypes (phase1_release_v3.20101123 without singletons and the minimac 2012-11-16 release; HIBAG v1.2.4 was used for imputing HLA genotypes (Zheng et al 2014). Standard quality control exclusions were applied.

### WUSTL

Genotyping was performed using the Illumina 610 or Omniexpress chip (Illumina, San Diego, CA, USA). QC removed variants with minor allele frequency <1%, genotyping rates <98%, Hardy–Weinberg equilibrium *P* values >10-6 and individuals with >2% missing genotypes. TOPMed Imputation Server (https://imputation.biodatacatalyst.nhlbi.nih.gov/#!) was used for phasing and imputation of nongenotyped.

**Acknowledgments S1. Acknowledgement List For ADNI Publications**

This file contains the full list of ADNI investigators.

## Supplementary Bibliography

Williams AL, Patterson N, Glessner J, Hakonarson H, Reich D. Phasing of many thousands of genotyped samples. Am J Hum Genet. 201210;91(2):238-251. doi: 10.1016/j.ajhg.2012.06.013.

Zheng X, Shen J, Cox C, Wakefield JC, Ehm MG, Nelson MR, Weir BS. HIBAG--HLA genotype imputation with attribute bagging. Pharmacogenomics J. 2014;14(2):192-200. doi: 10.1038/tpj.2013.18.
