# Supplementary material for: Patient stratification by genetic risk in Alzheimer’s disease is only effective in the presence of phenotypic heterogeneity
Source: PLoS One. 2025 Jan 9;20(1):e0310977. doi: 10.1371/journal.pone.0310977 (PMC11717250; doi:10.1371/journal.pone.0310977)
Supplement: S3 File — Full details on availability of data from each cohort can be found here. (DOCX) [file pone.0310977.s004.docx]

# *S3: Supplementary Detail on Data Availability*

Data for this manuscript was obtained from 4 cohorts, ADNI, NACC, Knight ADRC ("MAP") and an internal GSK cohort. Raw patient-level and genotype/phenotype data are available directly from the third-party databases as listed below. A summary of the full GWAS statistics are available on request to the authors.

ADNI Cohort: Full details of the cohort and steps for requesting access can be found at the following link: https://adni.loni.usc.edu/. Alongside the URL provided, the following DOI provides further details on the individual cohort used: 10.1016/j.jalz.2005.06.003. Permissions to use the data is granted to those who submit through the ADNI portal and sign the data use agreement. ADNI requires review of publications by the ADNI Data and Publications Committee prior to submission to ensure implementation of the required elements (ADNI listing in the author byline, citing as a data source, methods and acknowledgement of funding statements that pertain to ADNI data). The manuscript was shared with the ADNI committee and we received agreement from them that all required elements were included.

Knight ADRC Cohort: Full details of the cohort and steps for requesting access can be found at the following link: https://www.niagads.org/knight-adrc-collection. Alongside the URL provided, the following DOI provides further details on the individual cohort used: 10.3233/JAD-170834. Permissions to use the data have been obtained through Washington University (per agreement WUSTL Section 10.6), which is contracted to use data from the Knight ADRC Cohort and the data are available on request through the link.

NACC Cohort: Full details of the cohort and steps for requesting access can be found at the following link: https://naccdata.org/requesting-data/nacc-data. Alongside the URL provided, the following DOI provides further details on the individual cohort used: 10.1097/WAD.0b013e318142774e. Permissions to use the data have been obtained by signing the user agreement, referencing the NACC data and uploading of the publication to the NACC website for review.

The GSK Cohort was published in the original paper (Harrington et al 2011) can be found at the following link: https://pubmed.ncbi.nlm.nih.gov/21592048/. At this time, patient data is not available on request.
