# Supplementary material for: Patient stratification by genetic risk in Alzheimer’s disease is only effective in the presence of phenotypic heterogeneity
Source: PLoS One. 2025 Jan 9;20(1):e0310977. doi: 10.1371/journal.pone.0310977 (PMC11717250; doi:10.1371/journal.pone.0310977)
Supplement: S4 File — (DOCX) [file pone.0310977.s005.docx]

## Figure S1. GWAS in individual cohorts.

| A  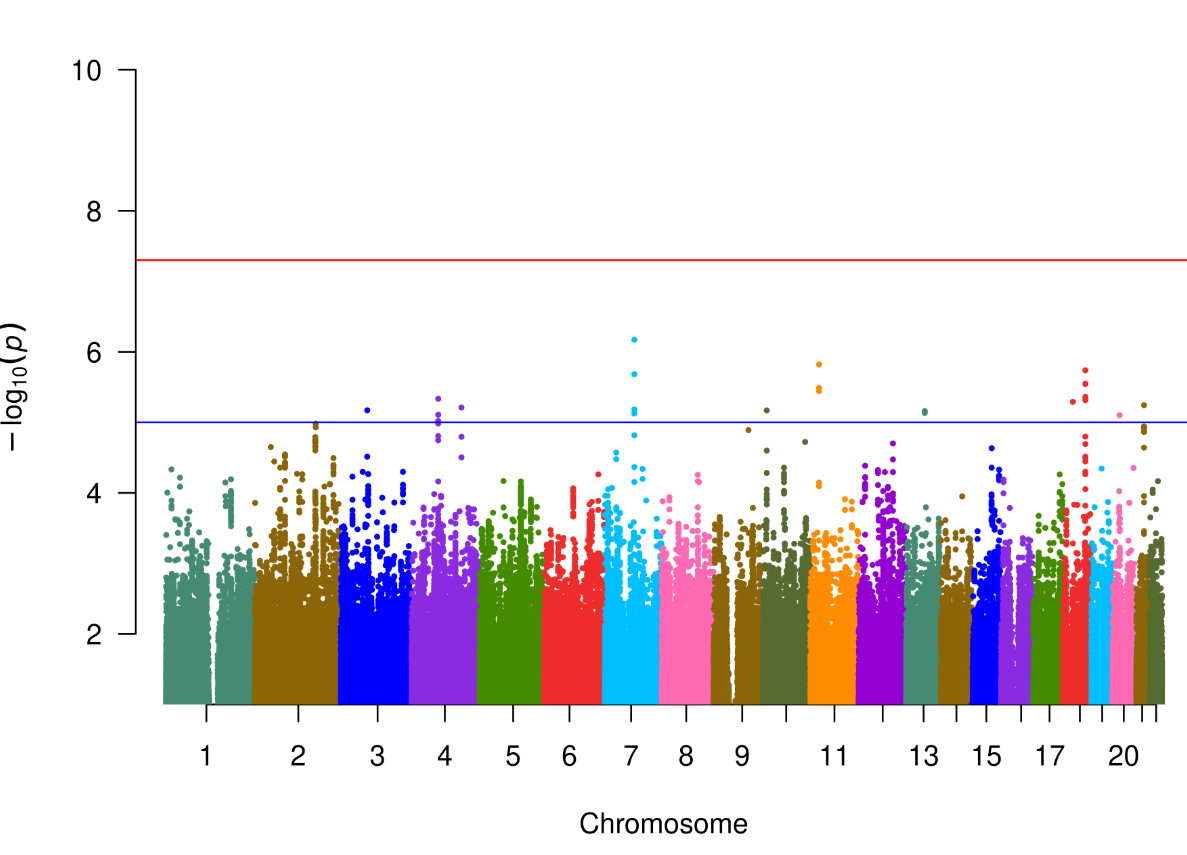 | B  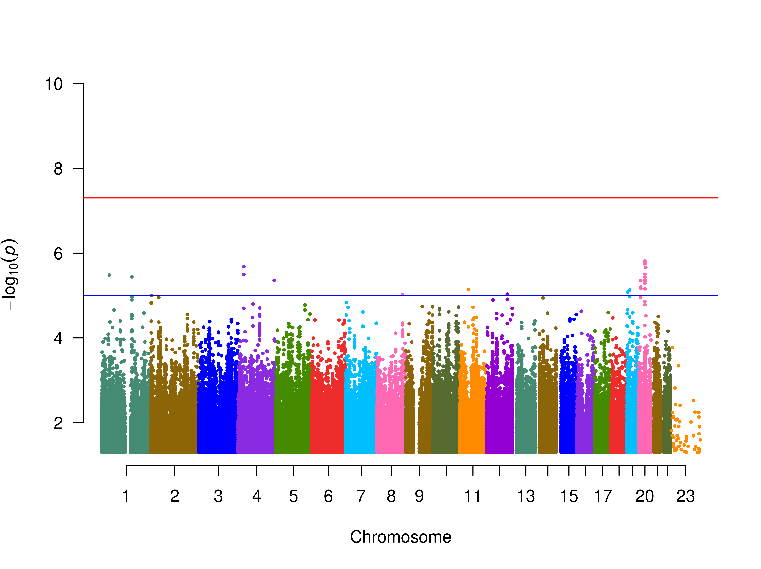 |
| --- | --- |
| C  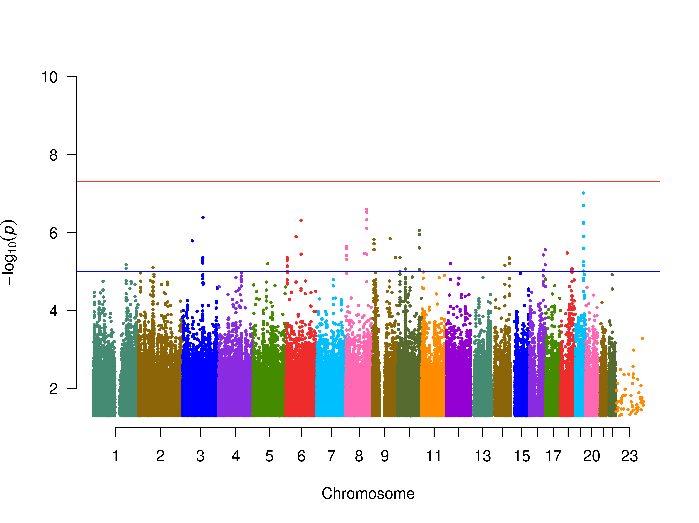 | D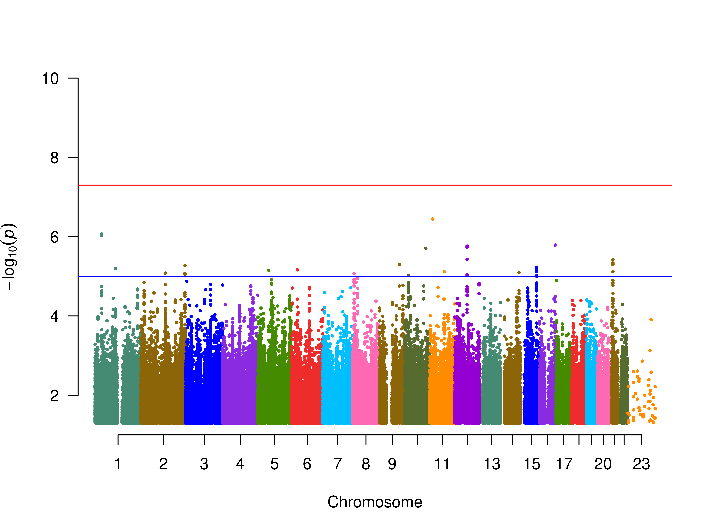 |

## Figure S2. Spaghetti plots by cohort.

| 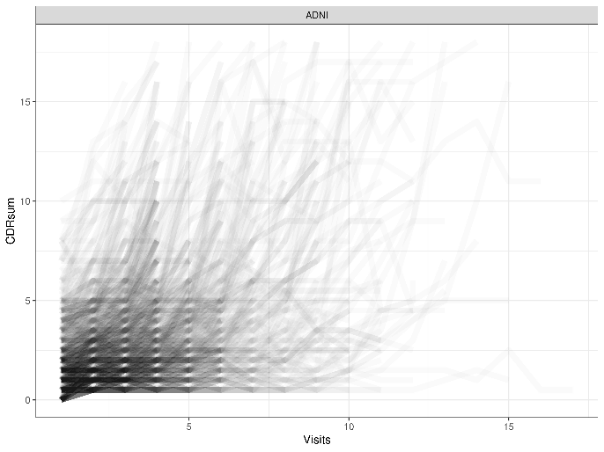 | 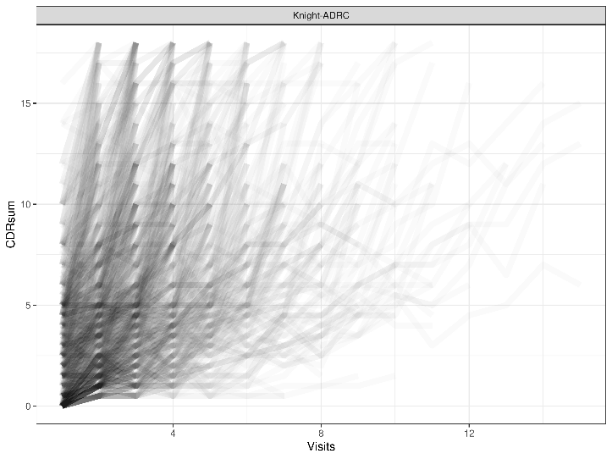 |
| --- | --- |
| 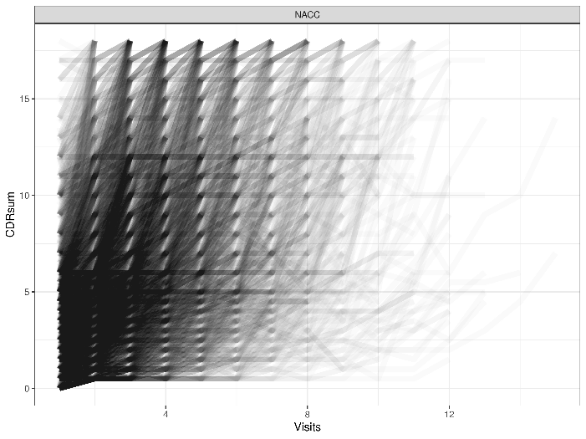 | 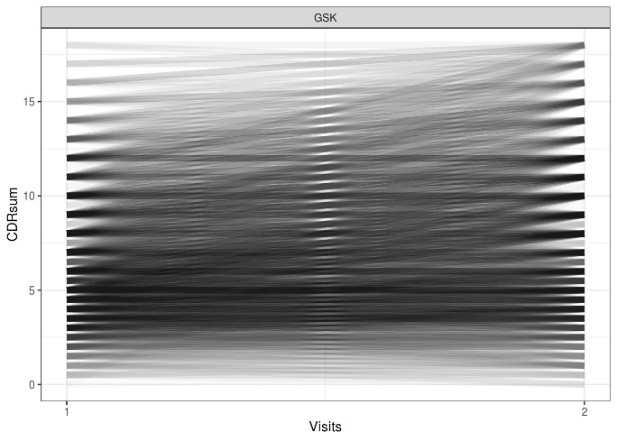 |

## Figure S3. Forrest plot of APOE-region SNP.


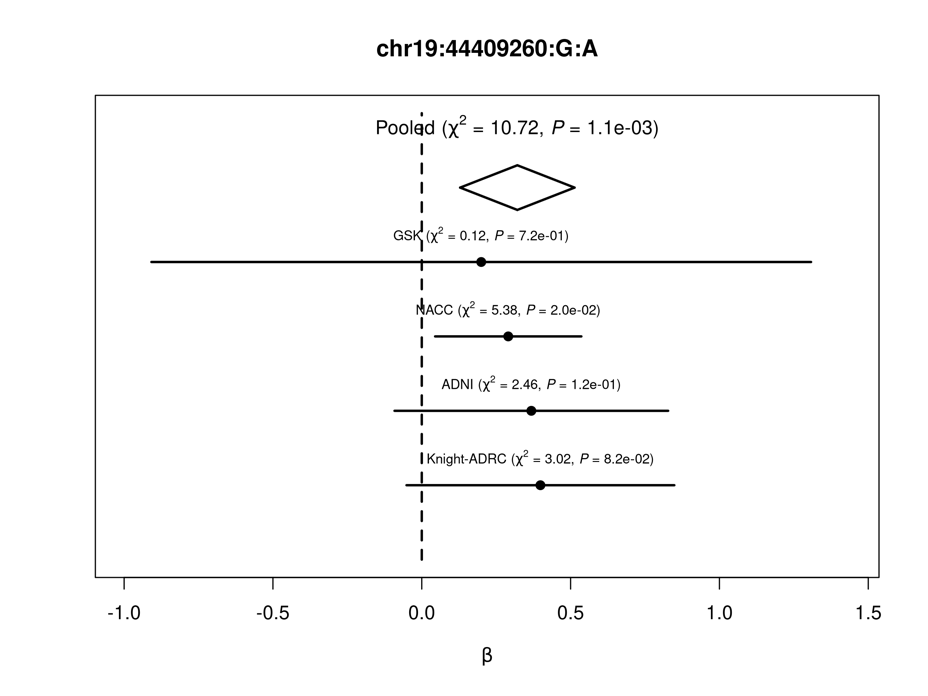


*Supplementary Figure 4***. Histogram of Phenotype per-cohort**

| 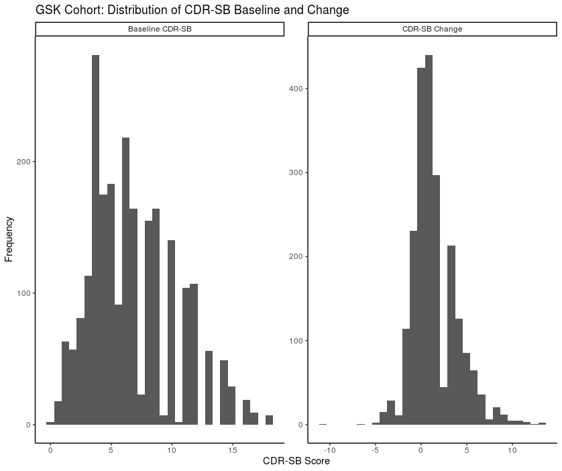 |
| --- |
| 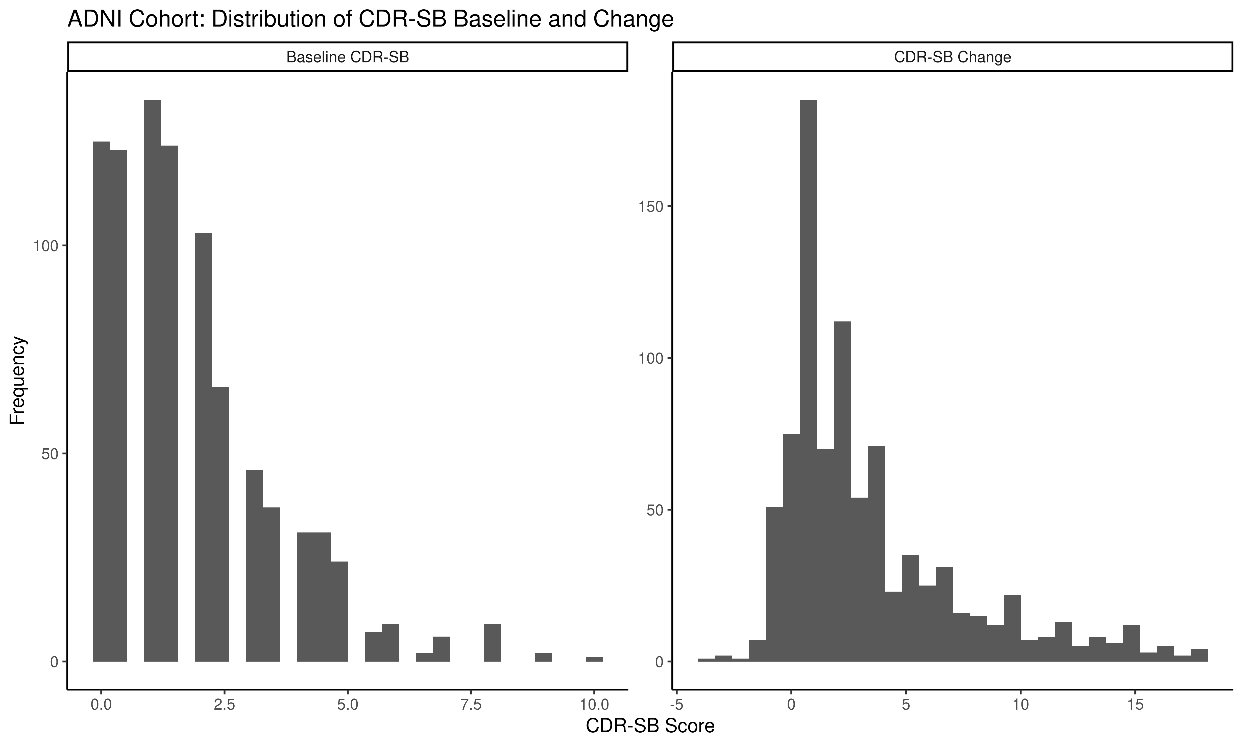 |
| 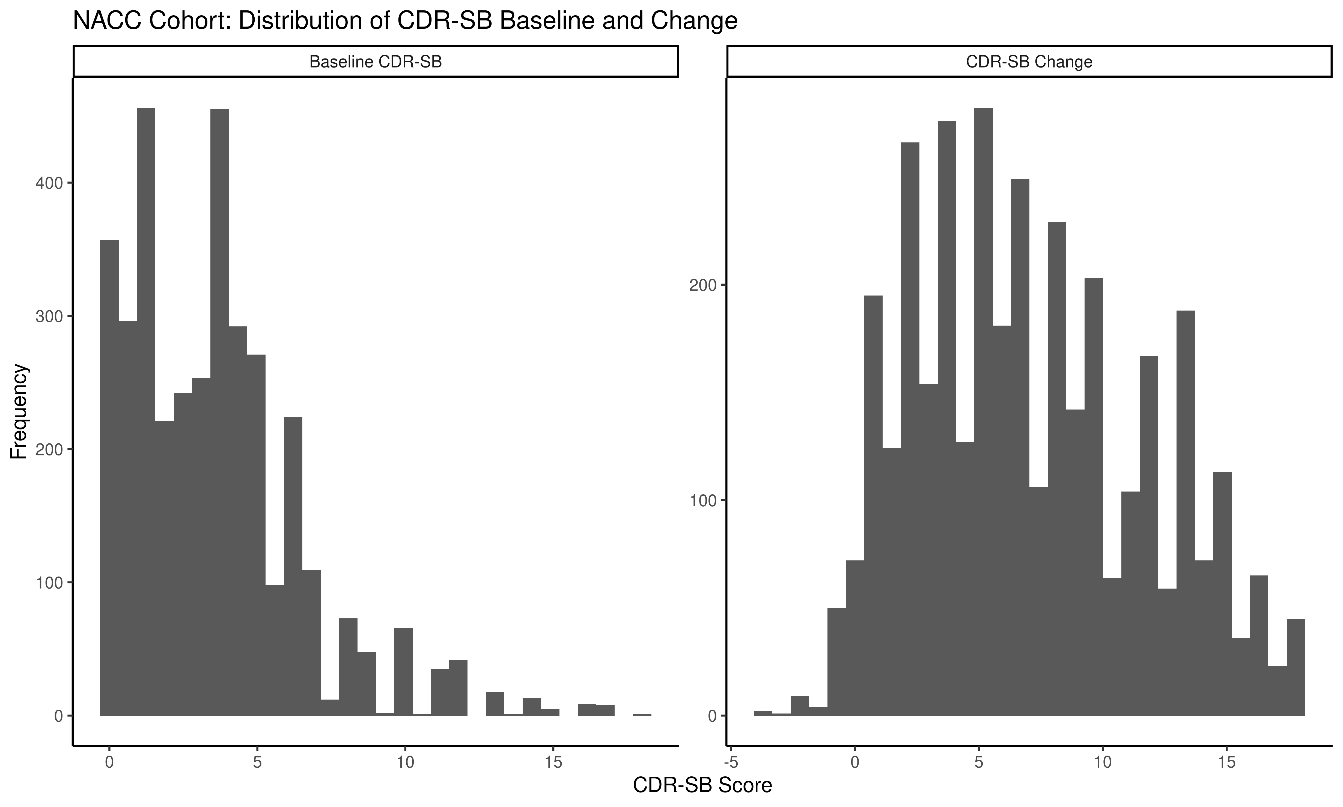 |
| 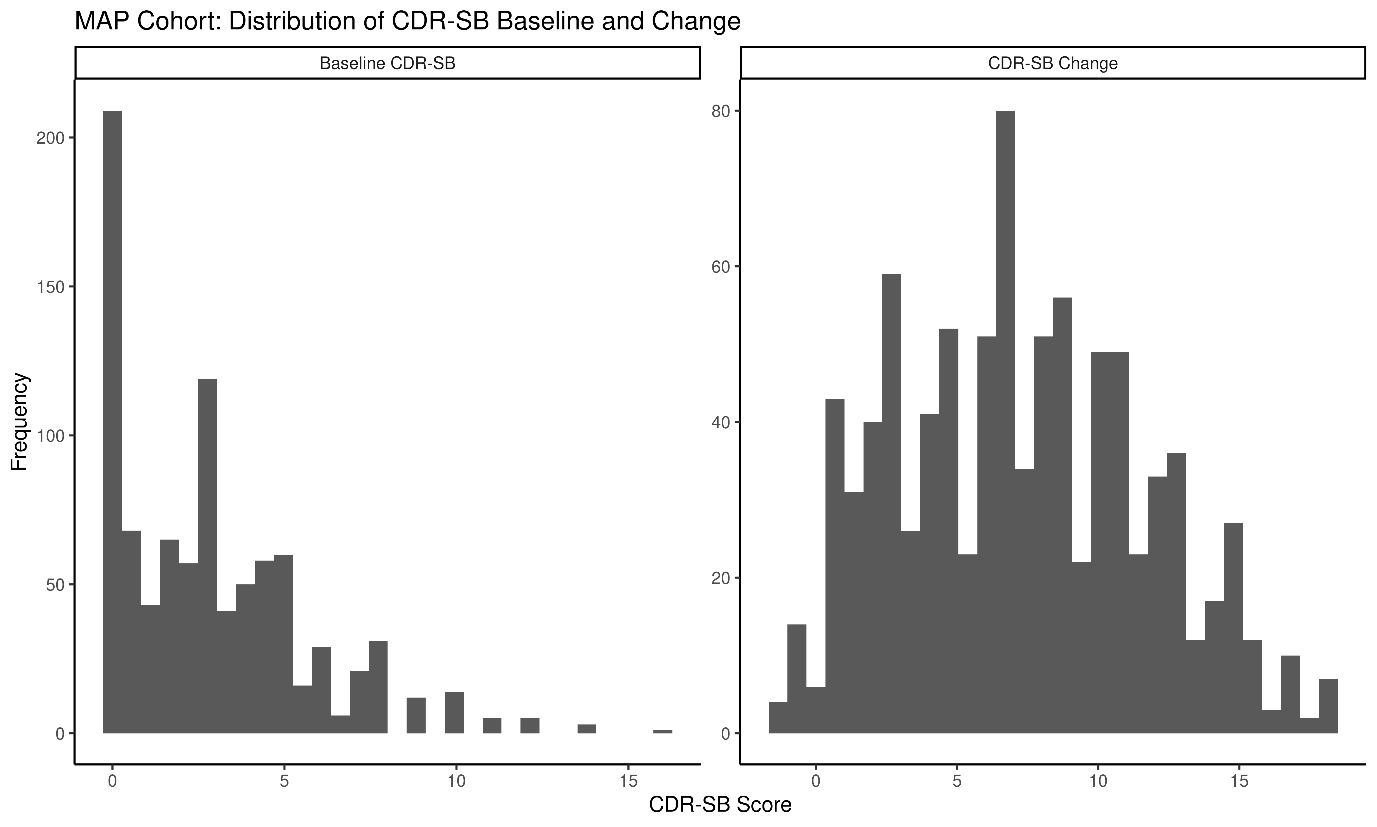 |
